# Patients with schizophrenia and bipolar disorder are characterized by different blood RNA editing signatures

**DOI:** 10.1192/j.eurpsy.2025.292

**Published:** 2025-08-26

**Authors:** D. Weissmann, F. J. C. Robles, N. Salvetat, C. Cayzac, M. Menhem, D. Vetter, I. Ouna, J. N. Nani, M. A. Hayashi, E. Brietzke

**Affiliations:** 1 Parc Euromédecine, ALCEDIAG, Montpellier, France; 2 Escola Paulista de Medicina (EPM), Universidade Federal de São Paulo (UNIFESP), Department of Pharmacology, SP; 3 National Institute for Translational Medicine (INCT-TM, CNPq/FAPESP/CAPES), Ribeirão Preto, Brazil; 4 Queen’s University School of Medicine, Department of Psychiatry, Kingston, Canada

## Abstract

**Introduction:**

Mental disorders, such as Bipolar Disorder (BD), Schizophrenia (SZ), and Schizoaffective Disorder (SA), are prevalent and often debilitating conditions that significantly impact individuals’ lives (Scangos et al. Nat Med 2023; 29(2): 317-33). Recent findings have identified blood RNA editing gene modifications that may aid in distinguishing between healthy controls, depressed patients, and those with BD and unipolar depression, improving diagnostic accuracy and treatment strategies (Salvetat et al. Transl Psychiatry 2022; 12(1):182).

**Objectives:**

This study demonstrates that RNA editing biomarkers can accurately differentiate individuals with SZ, SA, BD, and healthy controls, highlighting the potential of artificial intelligence (AI)-based predictions for diagnosis.

**Methods:**

A comparative analysis was performed with 85 healthy controls subjects, 39 BD, 31 SZ, and 14 SA patients. Patient samples were collected from two cohorts. Diagnostic assessments were conducted using SCID-1, HDRS, YMRS, and M.I.N.I., while healthy controls had no history of mental disorders or psychotropic medication use.

**Results:**

Significant biomarkers were combined using a multiclass Random Forest algorithm. The algorithm was trained on 70% of the population. Then, the test was performed on the 30% of the population who never saw the algorithm. The analysis shows clear differentiation between the control group and individuals with BD, SZ, and SA with high sensitivities and specificities for ROC area under the curve (AUC).

**Conclusions:**

This proof-of-concept analysis provides strong evidence for using RNA editing signature in diagnosis, and potentially in prognosis and treatment prediction. Further validation will be performed using a larger cohort.

**Disclosure of Interest:**

None Declared

